# The role of increased body mass index in outcomes of sepsis: a systematic review and meta-analysis

**DOI:** 10.1186/s12871-017-0405-4

**Published:** 2017-08-31

**Authors:** Sicong Wang, Xu Liu, Qixing Chen, Can Liu, Changshun Huang, Xiangming Fang

**Affiliations:** 1 Department of Anesthesiology and Intensive Care Medicine, the First Affiliated Hospital of Zhejiang University School of Medicine, Hangzhou, 310003 China; 2 Department of Intensive Care Medicine, the Affiliated Hospital of Guizhou Medical University, Guizhou, 550000 China; 3 Clinical Research Center, Children’s Hospital, School of Medicine, Zhejiang University, Hangzhou, 310052 China; 4 Department of Anesthesia, Ningbo First Hospital, Ningbo, 315010 China

**Keywords:** Sepsis, Body mass index, Obesity, Mortality, Length of stay

## Abstract

**Background:**

The role of increased body mass index (BMI) in sepsis is controversial. We aimed to evaluate the associations between overweight (25 kg/m^2^ < BMI ≤ 29.9 kg/m^2^), obese (30 kg/m^2^ < BMI ≤ 39.9 kg/m^2^) and morbidly obese (BMI > 40 kg/m^2^) BMIs and outcomes in septic patients.

**Methods:**

We searched the PubMed, Embase, Web of Science, Cochrane Library and ClinicalTrials.gov databases for studies published by December 1, 2016. Electronic database searches yielded 3713 articles, eight of which were included in this meta-analysis. Data were independently extracted by two reviewers, and a third reviewer participated in making decisions as needed. We used Review Manager to conduct the analysis, and the outcomes were reported with odds ratios (ORs) or mean differences (MDs). The primary outcome was mortality, and the secondary outcome was length of stay (LOS) in the intensive care unit (ICU) or the hospital.

**Results:**

Data from eight studies involving a total of 9696 patients were pooled in our final analysis. Compared with patients with normal BMI (18.5 kg/m^2^ < BMI ≤ 24.9 kg/m^2^), patients with BMI ≥ 25 kg/m^2^ exhibited decreased mortality (OR 0.81; 95% confidence interval (CI), 0.74–0.89, *P* < 0.0001). In subgroup analysis, compared with normal-weight patients, overweight patients had lower mortality (OR 0.87; 95% CI 0.77–0.97, *P* = 0.02), whereas obese (OR 0.89, 95% CI 0.72–1.10, *P* = 0.29) and morbidly obese (OR 0.64, 95% CI 0.38–1.08, *P* = 0.09) patients did not exhibit significantly reduced mortality.

**Conclusions:**

In sepsis cases, overweight, but not obesity or morbid obesity, was associated with lower mortality. Further prospective studies are needed to clarify this relationship.

**Electronic supplementary material:**

The online version of this article (doi:10.1186/s12871-017-0405-4) contains supplementary material, which is available to authorized users.

## Background

Sepsis occurs when an infection does not remain limited to local tissue but instead induces a series of dysregulated host responses that result in life-threatening organ dysfunction [1, 2]. Sepsis is one of the leading causes of death in intensive care units (ICUs), and sepsis and septic shock are important healthcare problems. Walkey et al. conducted a nationwide retrospective cohort study that identified 53.9 million adult infection hospitalizations from 2003 to 2009 and found that in the USA, the sepsis incidence rate has increased to 535 cases per 100,000 person-years and continues to rise [3]. Based on case fatality rates for prior decades, sepsis may cause or contribute to up to 5.3 million deaths worldwide each year [4, 5].

Global prevalence of overweight and obesity in children and adults from 1980 to 2013 were estimated by Ng et al., who found large increases in the prevalence over time [6]. Overweight and obesity are medical conditions in which excess body fat has accumulated to such an extent that it may negatively influence health. Studies have shown that obesity is associated with the pathogenesis and prognosis of myriad diseases, such as diabetes, hypertension, and cancer [7, 8]. However, various recent studies have demonstrated mixed results regarding the role of obesity in diseases. Notably, certain clinical studies addressing the effects of obesity on critical illnesses (such as heart failure, acute coronary syndrome and acute respiratory distress syndrome) have revealed an “obesity paradox” in which obesity is not harmful and can even be protective, including for patients who have already become sick [9–15].

The role of obesity in patient outcomes in specific ICU populations, such as septic patients, has been paid much attention; however, extant clinical data on this topic remain controversial. It is uncertain whether obesity could influence the acute risk of death in sepsis. Studies have indicated that obesity is correlated with an increased risk of death [16], whereas other investigations have reported inverse [17–19] or null [20–25] associations between obesity and risk of death. Two systematic reviews have analysed the effects of obesity in septic patients. However, a number of additional observational studies on the associations between body mass index (BMI) and risk of death among septic patients have not been included in meta-analysis [18, 21–23]. In addition, these two reviews only focused on the outcome of death and did not examine the effects of BMI on length of stay (LOS) in the ICU or the hospital, which is a major cause of high medical expenses. To further understand this problem, we performed a meta-analysis to address the associations between obesity, which was measured using BMI, and outcomes in sepsis.

## Methods

### Data sources and searches

We systematically searched the PubMed, Embase, Web of Science, Cochrane Library and ClinicalTrials.gov databases for articles published by December 1, 2016. Database-specific search terms included the key words ‘obesity’, ‘sepsis’ and ‘outcome’. An example of our search strategy is provided in an Additional file 1: Appendix A. We performed searches with no language restrictions but reviewed only English papers. Reference lists of relevant articles and reviews were manually screened to identify additional studies.

### Study selection

Two investigators independently screened all articles that satisfied our inclusion criteria. Studies were included in our analysis if they (1) utilized prospective or retrospective observational study designs (excluding systematic reviews, letters, editorials and so on. The full excluding list is given in Additional file 1: Appendix B); (2) involved adult patients with sepsis, severe sepsis, or septic shock; (3) defined obesity using BMI; and (4) included a comparison of mortality in patients across two or more BMI categories as an outcome.

### Data extraction and quality assessment

The same two investigators independently extracted data, and a third investigator participated in discussions to make a final decision if there was disagreement. The following data were extracted: first author; year of publication; country or region; study design; participants and population; outcome measures; BMI categories; demographic characteristics (age and gender); comorbidities (diabetes mellitus); assessments of disease (Acute Physiology and Chronic Health Evaluation (APACHE) II score and Simplified Acute Physiologic Score (SAPS) II); and study results. The primary outcome was mortality. Different statistical approaches were used to assess mortality in the included studies; in order of preference, we used ICU mortality, hospital mortality, 28-day mortality and 60-day mortality. The secondary outcome was LOS in the ICU or the hospital, and we stratified based on LOS in subgroup analyses.

The Newcastle-Ottawa Quality Assessment Scale (NOS) was used to assess the quality of cohort studies in our analysis.

### Statistical analysis

We used Review Manager 5.3.5 (The Nordic Cochrane Centre, The Cochrane Collaboration, Copenhagen, 2014) to conduct statistical analyses. Outcomes were reported as odds ratios (ORs) with 95% confidence intervals (CIs) for mortality and as mean differences (MDs) with 95% CIs for LOS in the ICU and the hospital. Heterogeneity among studies was assessed statistically using the Cochran Q test (with *P* < 0.10 regarded as statistically significant) and the I^2^ index (with I^2^ > 50% indicating substantial heterogeneity). A fixed-effect model was chosen for the calculation of pooled effects if heterogeneity was low; otherwise, a random-effect model was used for such calculations.

## Results

### Literature search

**Fig. 1:**
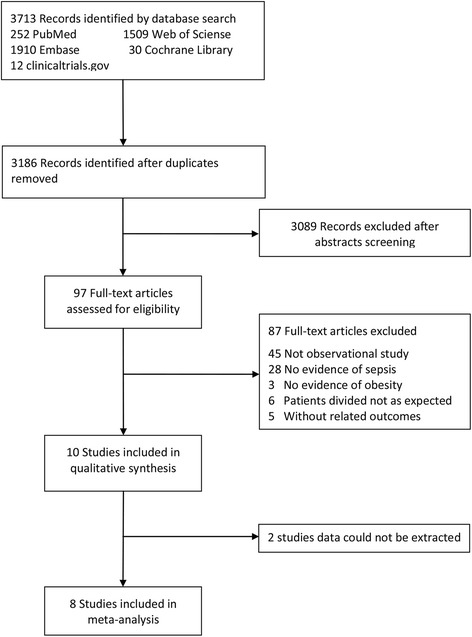
Flow diagram of study selection

We retrieved 3713 records from the database search, and Fig. 1 presents a flowchart that illustrates the study selection. After reviewing the titles and abstracts, we excluded 3089 studies. We checked the full text of the remaining 97 articles, and 10 of these articles satisfied our inclusion criteria. Two studies that satisfied our inclusion criteria was excluded because we could not extract needed data despite attempts to contact the corresponding authors via email to obtain the complete set of original data from these studies [26, 27]. Ultimately, 8 observational studies were included in our meta-analysis. No additional studies were found by screening reference lists.

### Basic characteristics of the included studies

The including studies, which involved 9696 patients, consisted of 6 retrospective cohort studies [18–22, 24] and 2 prospective cohort studies [23, 25]. All eight studies examined the effects of BMI on mortality in adult septic patients. The characteristics of the included studies are presented in Table 1. Four studies reported hospital LOS [20–22, 25], whereas only three studies reported ICU LOS [18, 20, 25]. Among the included studies, two studies reported a significant association between obesity and improved mortality [18, 19], three studies indicated that obesity was not related to mortality [23–25], and the remaining studies showed that obesity was protective but that the effect of obesity became non-significant after adjusting for baseline characteristics [20–22]. Compared with patients in other BMI categories, underweight patients experienced poor outcomes in all studies that used this category [20–22, 25]. Given this relatively consistent result; we excluded this category from our meta-analysis. Patients in six studies [18, 20–23, 25] were divided according to National Institutes of Health (NIH) criteria into multiple BMI categories; in the remaining two studies [19, 24], patients with BMI < 25 kg/m^2^ were compared with patients with BMI ≥ 25 kg/m^2^. The majority of patients enrolled in the included studies were men, and the mean age of subjects ranged from 50.5 to 81.3 years. Details of septic patients’ baseline characteristics in each study included in this analysis are shown in Table 2.

**Table 1:** Included studies and their main characteristics

First author, year	Country or region	Study type	Sample size	Diagnostic criteria	Outcome
Arabi [20], 2013	Canada, United States, and Saudi Arabia	retrospective study, multicentre	2882	septic shock	ICU mortality hospital mortality ICU LOS hospital LOS
Chalkias [23], 2013	Greece	prospective study, single-centre	30	sepsis	60-day mortality morbidity, length of ventilation in ICU.
Gaulton [22], 2015	USA	retrospective study, single-centre	1191	severe sepsis	28-day mortality 60-day mortality 1-year mortality hospital LOS
Kuperman [21], 2013	USA	retrospective study, single-centre	792	sepsis	inpatient mortality hospital LOS
Pisitsak [24], 2016	Canada	retrospective study, multicentre	257	sepsis	28-day mortality 90-day mortality
Prescott [18], 2014	USA	retrospective study, multicentre	1404	severe sepsis	hospital mortality 90-day mortality 1-year mortality ICU LOS
Sakr [25], 2008	Europe	prospective study, multicentre,	2978	sepsis	ICU mortality hospital mortality ICU LOS hospital LOS
Wacharasint [19], 2013	Canada	retrospective study, multicentre	730	septic shock	28-day mortality

*BMI* body mass index; *ICU* intensive care unit; *LOS* length of stay

**Table 2:** Baseline characteristics of patients in each trial included in the analysis

Author, year	BMI (kg/m^2^) categories	Sample size	Age years, mean (SD)	Male, *n* (%)	APACHE II, mean(SD)	SAPS II, mean (SD)	Diabetes mellitus, n (%)	Mortality data included in the analysis *n* (%)	Mean (SD) or median(range)	
									Hospital LOS	ICU LOS
Arabi [20], 2013	underweight(<18.5)	196	59.1 (19.2)	114 (58.2%)	25.5 (8.0)	NR	11 (5.6)	102 (52.0%)	25.1 (33.6)	9.9 (9.4)
	normal(18.5–24.9)	1020	62.2 (16.8)	630 (61.8%)	25.7 (8.1)		76 (7.5)	451 (44.2%)	25.1 (31.8)	10.5 (12.5)
	overweight(25.0–29.9)	816	63.5 (15.9)	500 (61.3%)	25.6 (8.4)		82 (10.1)	335 (41.1%)	27.8 (35.2)	11.4 (14.7)
	obese (30.0–39.9)	680	62.2 (14.6)	346 (50.9%)	25.4 (7.9)		80 (11.8)	265 (39.0%)	26.5 (34.8)	11.2 (14.3)
	morbidly obese(>40.0)	170	58.4 (13.0)	68 (40.0%)	24.4 (7.3)		35 (20.6)	57 (33.5%)	34.3 (44.2)	12.2 (12.7)
Chalkias [23], 2013	normal(18.5–24.9)	14	NR	NR	NR	NR	NR	2 (6.9%)	NR	NR
	overweight(25.0–29.9)	7						0		
	obese(30.0–39.9)	8						3 (10.3%)		
Gaulton [22], 2015	underweight(<18.5)	102	57.5 (19.3)	53 (52%)	15 (7.4)	NR	14 (13.7%)	24 (23.5%)	8 (4–14)	NR
	normal(18.5–24.9)	480	57 (19.3)	271 (56.5%)	15 (7.4)		97 (20.2%)	105 (21.9%)	6 (4–11)	
	overweight(25.0–29.9)	301	58 (17.0)	189 (62.8%)	14 (6.7)		79 (26.3%)	60 (19.9%)	7 (4–13)	
	obese(30.0–39.9)	229	56 (14.1)	112 (48.9%)	15 (5.2)		81 (35.4%)	43 (18.8%)	6.5 (4–14)	
	morbidly obese(>40.0)	79	50.5 (15.6)	26 (34.2%)	12 (7.4)		23 (29.1%)	5 (6.3%)	5 (3–9.5)	
Kuperman [21], 2013	underweight(<18.5)	49	60.3 (22.9)	17 (34.7%)	16.2 (6.6)	NR	5 (10%)	12 (24.5%)	8.1 (7.2)	NR
	normal(18.5–24.9)	261	61.2 (19.2)	155 (59.4%)	15.4 (5.8)		44 (17%)	46 (17.6%)	9.3 (8.5)	
	overweight(25.0–29.9)	249	64.2 (16.0)	144 (57.8%)	15.6 (5.8)		52 (21%)	40 (16.1%)	8.8 (7.8)	
	obese(30.0–39.9)	187	60.8 (14.2)	102 (54.5%)	15.9 (6.1)		65 (35%)	25 (13.4%)	9.0 (8.1)	
	morbidly obese(40.0–49.9)	46	60.2 (13.7)	12 (26.1)	14.2 (5.6)		22 (48%)	6 (13.0%)	10.2 (9.6)	
Pisitsak [24], 2016	BMI < 25	82	NR	NR	NR	NR	NR	22 (26.8%)	NR	NR
	BMI ≥ 25	175						41 (23.4%)		
Prescott [18], 2014	normal(18.5–24.9)	597	81.3 (8.6)	277 (46.6%)	NR	NR	62 (10.4%)	161 (34.0%)	NR	10.4 (9.6)
	overweight(25.0–29.9)	473	78.7 (8.6)	274 (57.9%)			94 (19.9%)	96 (47.5%)		11.5 (12.0)
	obese(30.0–34.9)	202	75.3 (8.1)	93 (46.0%)			48 (23.8%)	36 (27.3%)		11.5 (10.3)
	Severelyobese (>35.0)	132	72.8 (8.0)	37 (28.0%)			31 (23.5%)	24 (18.2%)		12.5 (13.3)
Sakr [25], 2008	underweight(<18.5)	120	52.6 (21)	55 (46.8%)	NR	32.3 (15.8)	6 (5%)	23 (19.2%)	11.8 (4.1–22.3)	2.8 (1.4–6.4)
	normal(18.5–24.9)	1206	58.4 (19.1)	739 (61.9%)		36.3 (16.8)	75 (6.2%)	216 (17.9%)	12.3 (5.1–24.4)	3.1 (1.7–7.2)
	overweight(25.0–29.9)	1047	63.6 (15)	712 (68.8%)		36.8 (17.1)	80 (7.6%)	181 (17.3%)	11.1 (5.5–24.5)	3.1 (1.7–7.0)
	obese(30.0–39.9)	424	63.5 (14.1)	238 (56.8%)		37.6 (17.7)	34 (8%)	84 (19.8%)	12.2 (5.8–24.2)	3.6 (1.8–7.1)
	morbidly obese(>40)	81	56.9 (15)	31 (38.3%)		35.7 (17.8)	10 (12.3%)	16 (19.8%)	14.3 (8.4–27.4)	4.1 (1.8–12.1)
Wacharasint [19], 2013	BMI < 25	276	63 (19.3)	171 (62%)	27 (8.1)	NR	41 (14.9)	114 (41.3%)	NR	NR
	25 < BMI < 30	209	64 (17.0)	142 (67.9%)	26 (6.7)		43 (20.6)	74 (35.4%)		
	BMI > 30	245	63 (14.1)	136 (55.5%)	27 (6.7)		73 (29.8)	71 (29.0%)		

*BMI* body mass index; *ICU* intensive care unit; *LOS* length of stay; *NR* not reported

The NOS was used to assess the quality of individual studies. The maximum total score on this scale, which summarizes eight aspects of each study, was 9 points. A study with a final score ≥ 6 was regarded as a high-quality investigation. All of the included studies exhibited high quality (Additional file 1: Appendix C).

### Mortality

**Fig. 2:**
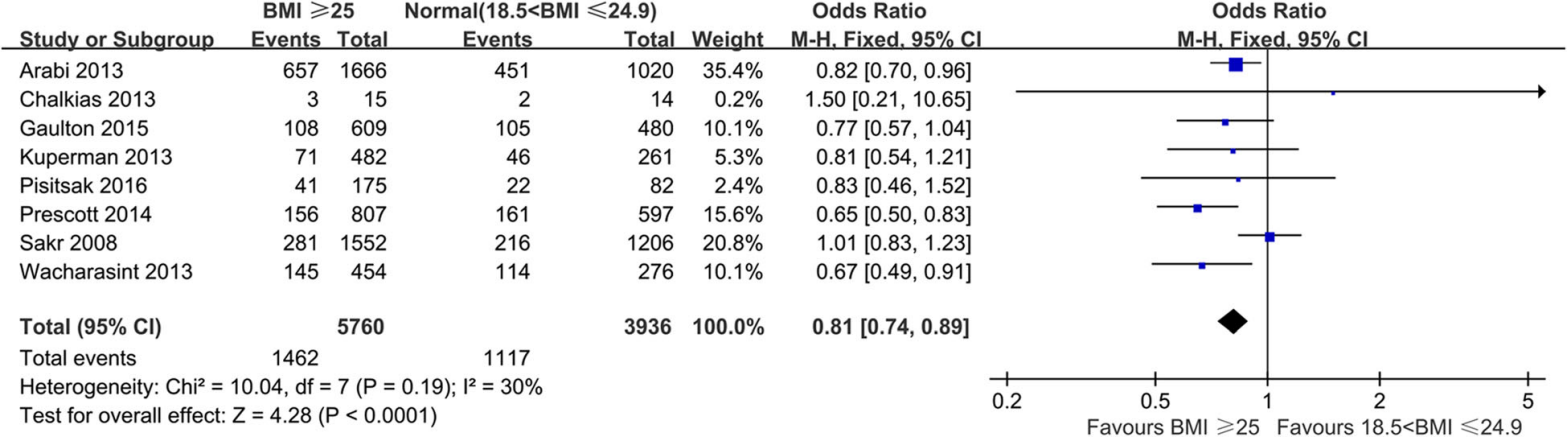
Meta-analysis of mortality between BMI ≥ 25 kg/m^2^ and normal (18.5 kg/m^2^ < BMI ≤ 24.9 kg/m^2^) septic patients

**Fig. 3:**
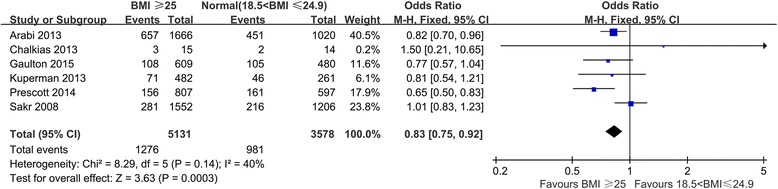
Meta-analysis of mortality between BMI ≥ 25 kg/m^2^ and normal (18.5 kg/m^2^ < BMI ≤ 24.9 kg/m^2^) septic patients after excluding two studies with considerable heterogeneity

**Fig. 4:**
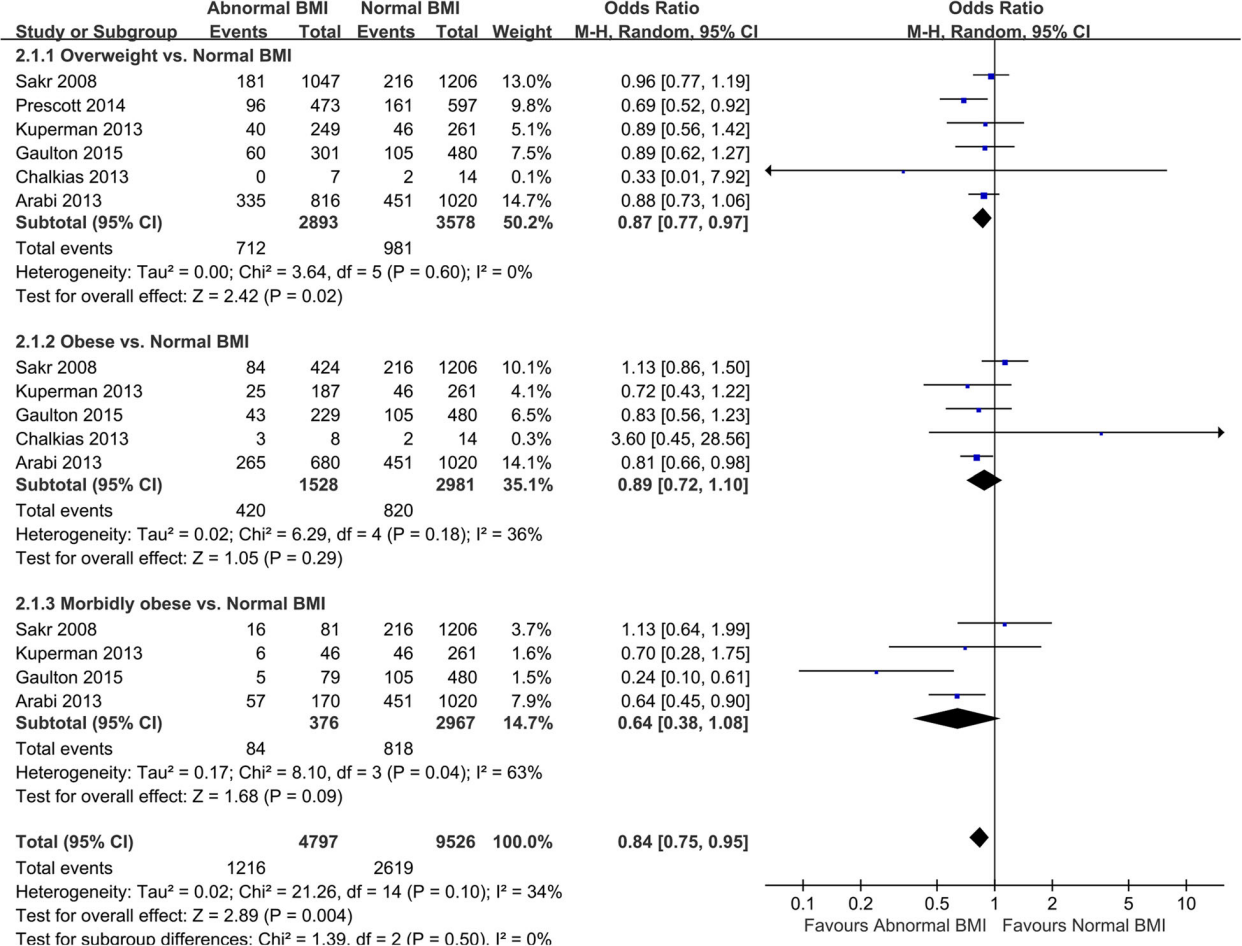
Subgroup meta-analysis of the impact of BMI on mortality

We pooled data from the eight included studies, which provided mortality results for patients with BMI ≥ 25 kg/m^2^ and normal-weight patients, and found that relative to other subjects, patients with BMI ≥ 25 kg/m^2^ exhibited decreased mortality (OR 0.81; 95% CI 0.74–0.89, *P* < 0.0001) (Fig. 2). We removed two studies because they included underweight patients in the reference [19, 24], conducted the same comparison again, and found that the trend of decreased mortality among patients with high BMI remained statistically significant (OR 0.83; 95% CI 0.75–0.92, *P* = 0.0003) (Fig. 3). We extracted data from studies that provided case fatality information and compared patients in different BMI categories (Fig. 4). Overweight patients had lower mortality than normal-weight patients (OR 0.87; 95% CI, 0.77–0.97, *P* = 0.02). Although the observed differences were not statistically significant, similar trends of decreased mortality in pooled estimates for obese (OR 0.89, 95% CI, 0.72–1.10, *P* = 0.29) and morbidly obese (OR 0.64, 95% CI 0.38–1.08, *P* = 0.09) subjects relative to normal-weight reference patients were observed.

### ICU and hospital LOS

**Fig. 5:**
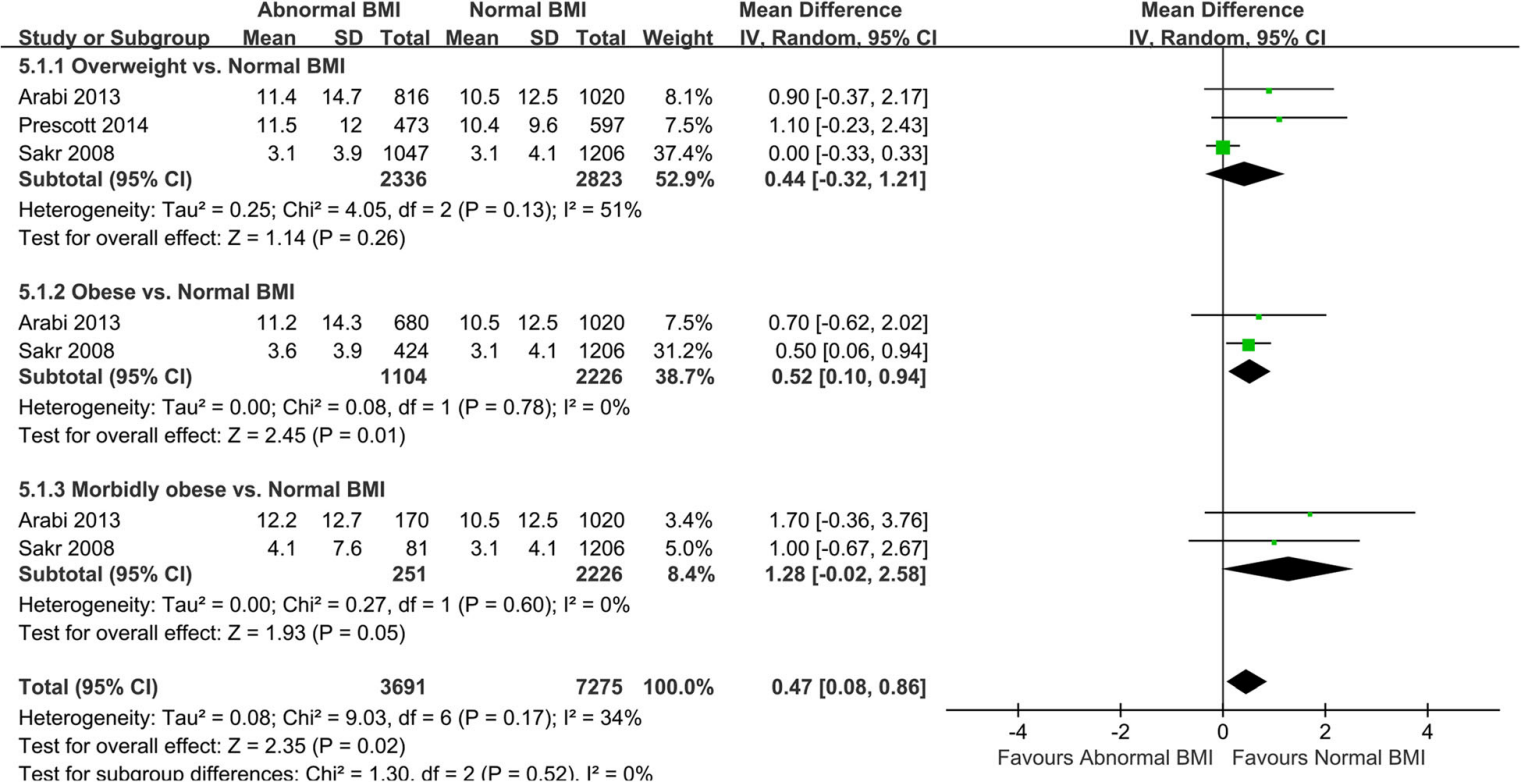
Subgroup meta-analysis of the impact of BMI on ICU LOS

ICU stay was shorter for obese patients than for normal-weight patients (MD 0.52, 95% CI 0.10–0.94, Z = 2.45, *P* = 0.01) but did not significantly differ between obese and morbidly obese patients (Fig. 5). With respect to hospital LOS, overweight (MD 0.19, 95% CI -1.21-1.59, Z = 0.26, *P* = 0.79), obese (MD 0.22, 95% CI -0.52-0.96, Z = 0.59, *P* = 0.56), and morbidly obese patients (MD 1.47, 95% CI -1.37-4.32, Z = 1.02, *P* = 0.31) did not significantly differ from normal-weight patients (Additional file 1: Appendix D).

### Publication bias

We were unable to assess publication bias due to the small number of studies included in each comparison [28].

## Discussion

This meta-analysis showed that patients with BMI ≥ 25 kg/m^2^ exhibited decreased mortality compared with that of normal-weight patients, which is an example of the “obesity paradox” and can be explained in several ways. First, sepsis is an acute illness involving a highly catabolic state, and excess body fat may serve as a source of fuel and energy during the course of this disease. In particular, research has indicated that higher body weight provides a nutritional reserve that becomes important for survival during an acute life-threatening illness [14]. Adipocytes store excess calories as triglycerides and release fuel (as fatty acids and glycerol) for use by other organs in times of caloric need [29]. Second, adipose tissue can also regulate immunity by excreting proteins such as leptin, which is an anti-inflammatory adipokine [30]. Obese individuals have elevated leptin levels at baseline. These levels are regulated by several factors; for instance, leptin can be increased by acute infection and proinflammatory cytokines. Bornstein et al. found that in acute sepsis cases, mean plasma leptin levels are threefold higher in survivors than in non-survivors; they assumed that leptin may play a role in a severe stress state such as acute sepsis [31]. In an animal study, researchers used a long-term cecum ligation and puncture model of sepsis with feeding of a high-fat diet to investigate the effects of obesity on immune function and outcome in sepsis. They showed that relative hyperleptinaemia induced by obesity or treatment is protective in sepsis, possibly via stabilizing body temperature, improving cellular immune response, and reducing proinflammatory cytokine response. Leptin appears to play a regulatory role in the immune system in sepsis [32]. Levels of adiponectin, an anti-inflammatory adipokine, change during the course of sepsis. In sepsis, higher adiponectin levels before sepsis and decreasing adiponectin levels after sepsis are associated with survival. Additionally, compared with patients who survived sepsis, O’Brien et al. found lower adiponectin concentrations before sepsis in the subgroup of patients who died during the course of sepsis [33]. Finally, obesity may be treated more aggressively than other diseases, in part because it is associated with high risks of cardiovascular disease, insulin resistance, hypertension, and other comorbidities [34]. Recent studies showed that treatment of severe sepsis differs according to BMI [35, 36]. Authors have also observed that similar volumes of resuscitation fluids were administered to sepsis patients irrespective of BMI. Thus, obese patients received a lower volume/kg of such fluids than patients with normal BMI [20]. In sepsis, improved outcomes for obese patients may be associated with a relatively restrictive fluid management strategy [37–39]. Clinically, physicians caring for adult patients likely do not routinely consider weight and height during decision making. The same situation will arise with respect to the administration of vasopressin or antibiotics. The question of whether weight-based dosing (in terms of mcg/kg/min) is superior to non-weight-based dosing (in terms of mcg/min) for these drugs has not been definitively answered.

In subgroup analysis, we also found that overweight patients had lower mortality than normal-weight patients. Researchers in Denmark have demonstrated that for cohorts enrolled from 1976 to 1978 through 2003–2013, the BMI associated with the lowest all-cause mortality has increased over time to 27.0 kg/m^2^ [40]. In other words, people with BMI of 27.0 kg/m^2^, which is in the overweight range, may have the lowest risk of death; this result is consistent with our findings. However, significantly decreased mortality was not observed in obese or morbidly obese patients relative to normal-weight reference patients. Relatively few obese and morbidly obese patients (only 1528 and 376 patients, respectively) were included in our analysis; in addition, due to inherent limitations, existing clinical studies may not have detected acquired comparative survival advantages. For instance, obese and morbidly obese patients may have an inherited genetic susceptibility for developing sepsis and therefore exhibit a higher risk of death. However, the overweight population in these studies may be represented by individuals whose overall health is relatively good. Second, an unmeasured confounding variable in our meta such as smoking, which has been identified to be associated with mortality after severe sepsis, may also explain the aforementioned results [41]. On the other hand, body fat distribution and body composition may differ between overweight patients and obese or morbidly obese patients, resulting in different effects of excess weight on outcomes in sepsis. Researchers have shown that ectopic fat accumulation is strongly associated with higher metabolic disease risk. Interestingly, subcutaneous fat accumulation confers beneficial systemic metabolic effects, whereas visceral fat does not [42, 43]. Investigators have used the term “metabolically healthy obesity” to refer to the preferential deposition of fat in subcutaneous depots in a manner that maintains insulin sensitivity, lowers infiltration by inflammatory cells and involves adipocytes with relatively favourable adipokine expression profiles [44].

Our results can be compared with the findings from the two prior meta-analyses of obesity and mortality after sepsis. A systematic review without analysis included seven studies with mixed results indicating that obesity might increase, reduce or not affect mortality [45]. Another meta-analysis examined the effect of different BMI categories on mortality after adjusting for other influential baseline variables and found that overweight or obese BMI was associated with reduced adjusted mortality in adults admitted to the ICU with sepsis, severe sepsis, or septic shock [46]. Our study included more valuable studies and performed further analyses. We found that patients with BMI ≥ 25 kg/m^2^ exhibited decreased mortality. Additionally, the subgroup analysis demonstrated that overweight was associated with lower mortality. Although we failed to observe a significant association between obese or morbidly obese BMI and decreased mortality, we also did not find obese or morbidly obese BMI to be correlated with worse outcome. Furthermore, we extracted available data, analysed the adjusted effects of overweight BMI on the OR for mortality relative to normal BMI, and showed that overweight BMI reduced adjusted mortality (Additional file 1: Appendix E). What’s more, we looked at hospital and ICU LOS. Despite these findings, more studies reporting the effects of BMI adjusted for other influential baseline variables are necessary due to the small quantity of currently available data, which may have resulted in bias.

Our study has several limitations. We focus on sepsis in our study but could not exclude the possibility that comorbidities such as diabetes mellitus or coronary heart disease may have improved or worsened outcomes [47]. Moreover, septic patients in hospitals are treated using different interventions, as described above; these differences introduce added complexity to the examined issue. Although the association between BMI and mortality was adjusted for age, gender and several other variables in certain studies [19, 20], residual confounders could not be considered and may have played a role in determining outcomes. We used BMI because it is relatively easy to determine and is widely used to measure degree of obesity in observational studies. However, BMI is not measured as consistently as expected. BMI data in the eight included studies are derived differently, including from a database, from self-reported information or from in-hospital measurements. Furthermore, inaccuracies can be introduced if weight is checked after fluid resuscitation. A recent study showed that BMI may not be an accurate predictor of risks associated with obesity. Chalkias et al. used sagittal abdominal diameter (SAD) to measure visceral obesity and found that increased SAD may predict future complications and increased risk for death in a BMI-independent manner [23]. A deeper understanding of obesity will allow us to further examine the impact of obesity on sepsis with better and more reliable standards.

## Conclusion

Compared with normal-weight patients, patients with BMI ≥ 25 kg/m^2^ exhibited decreased mortality. Overweight BMI is associated with lower mortality in patients with sepsis; moreover, obesity and morbid obesity were not associated with increased mortality. Further research is warranted to determine the pathophysiologic mechanisms underlying this observation. Moreover, more large studies, particularly well-designed prospective studies, are needed to further elucidate the precise roles of obesity in patients with sepsis. An understanding of the impact of obesity in septic patients may allow for accurate risk stratification and has implications for the development of prevention and treatment strategies.

## Additional file

Additional file 1: The role of increased body mass index in outcomes of sepsis: A systematic review and meta-analysis. (DOCX 385 kb)

## Abbreviations

95% CI: 95% confidence interval
APACHE: Acute Physiology and Chronic Health Evaluation
BMI: body mass index
ICU: intensive care unit
LOS: length of stay
MD: mean difference
NR: not reported
OR: odds ratio
SAPS: Simplified Acute Physiologic Score
