# Supplementary material for: The role of increased body mass index in outcomes of sepsis: a systematic review and meta-analysis
Source: BMC Anesthesiol. 2017 Aug 31;17:118. doi: 10.1186/s12871-017-0405-4 (PMC5579888; doi:10.1186/s12871-017-0405-4)
Supplement: Additional file 1: — The role of increased body mass index in outcomes of sepsis: A systematic review and meta-analysis. (DOCX 385 kb) [file 12871_2017_405_MOESM1_ESM.docx]

**Additional file**

APPENDIX A. Search strategy.

APPENDIX B. Full Excluding List.

APPENDIX C. Newcastle-Ottawa Quality Assessment Scale for studies.

APPENDIX D. Effect of BMI on ICU LOS.

APPENDIX E. Adjusted odds ratios for mortality in sepsis.

**APPENDIX A**

Search strategy

PubMed

(((("Sepsis"[Mesh]) OR ((((severe sepsis[Title/Abstract]) OR septic shock[Title/Abstract]) OR bacteremia[Title/Abstract]) OR septicemia[Title/Abstract]))) AND (("Obesity"[Mesh]) OR ((((((obese[Title/Abstract]) OR overweight[Title/Abstract]) OR "morbidly obese"[Title/Abstract]) OR "morbid obesity"[Title/Abstract]) OR BMI[Title/Abstract]) OR "body mass index"[Title/Abstract]))) AND ((((outcome*[Title/Abstract]) OR mortality[Title/Abstract]) OR Morbidity[Title/Abstract]) OR susceptibility[Title/Abstract]) Sort by: [relevance]

EMBASE

'sepsis'/exp OR 'severe sepsis':ab,ti OR 'septic shock':ab,ti OR 'bacteremia':ab,ti OR 'septicemia':ab,ti AND ('obesity'/exp OR 'obese':ab,ti OR 'overweight':ab,ti OR 'morbidly obese':ab,ti OR 'morbid obesity':ab,ti OR 'bmi':ab,ti OR 'body mass index':ab,ti) AND ('outcome*':ab,ti OR 'mortality':ab,ti OR 'morbidity':ab,ti OR 'susceptibility':ab,ti)

Cochrane Library Database

#1 MeSH descriptor: [Sepsis] explode all trees

#2 "severe sepsis":ti,ab,kw or "septic shock":ti,ab,kw or "bacteremia":ti,ab,kw or "septicemia":ti,ab,kw (Word variations have been searched)

#3 MeSH descriptor: [Obesity] explode all trees

#4 "obese":ti,ab,kw or "overweight ":ti,ab,kw or "morbidly obese":ti,ab,kw or "morbid obesity":ti,ab,kw or BMI:ti,ab,kw (Word variations have been searched)

#5 "body mass index":ti,ab,kw (Word variations have been searched)

#6 "outcome* ":ti,ab,kw or "mortality ":ti,ab,kw or "Morbidity ":ti,ab,kw or "susceptibility":ti,ab,kw (Word variations have been searched)

#7 #1 or #2

#8 #3 or #4 or #5

#9 #6 and #7 and #8

Web of Science

#1 TS= ("sepsis" OR “severe sepsis” OR “septic shock” OR “bacteremia” OR “septicemia”)

#2 TS= ("Obesity" OR “obese” OR “overweight” OR "morbidly obese" OR "morbid obesity" OR “BMI” OR "body mass index")

#3 TS=( “outcome*” OR “mortality” OR “Morbidity” OR “susceptibility”)

#4 #1 AND #2 AND #3

**APPENDIX B**

Full Excluding List

Two investigators independently screened all articles, then read 97 full-text articles in which 87 articles were finally excluded. The excluded articles are listed as follows:

**Not Observational Study: 45**

**Review: 22**

**Conference summary: 13**

**Case report: 3**

**Letter: 3**

**Editorials: 3**

**Poster presentation: 1**

**No evidence of sepsis: 28**

**No evidence of obesity: 3**

**Patients divided not as expected: 6**

**Without related outcomes: 5**

**Review: 22**

1. Esteban A, Frutos-Vivar F, Anzueto A. Impact of body weight on critically ill patients: a heavy load!! Intensive Care Med. 2008;34:1964-5.

2. Falagas ME, Athanasoulia AP, Peppas G, Karageorgopoulos DE. Effect of body mass index on the outcome of infections: a systematic review. Obes Rev. 2009;10:280-9.

3. Huttunen R, Karppelin M, Syrjänen J. Obesity and nosocomial infections. J Hosp Infect. 2013;85:8-16.

4. Savel RH, Simon RJ, Kupfer Y. Unraveling the Mysterious Relationship Between Obesity and Outcomes in Patients With Sepsis. Crit Care Med. 2016;44:2104-5.

5. Vachharajani V, Vital S. Obesity and sepsis. J Intensive Care Med. 2006;21:287-95.

6. Huttunen R, Syrjänen J. Obesity and the risk and outcome of infection. Int J Obes. 2013;37:333-40.

7. Rattan R, Nasraway SA Jr. Separating wheat from chaff: examining the obesity paradox in the critically ill. Crit Care. 2013;17:168.

8. Vachharajani V. Influence of obesity on sepsis. Pathophysiology. 2008;15:123-34.

9. Miehsler W. Mortality, morbidity and special issues of obese ICU patients. Wien Med Wochenschr. 2010;160:124-8.

10. Mittwede PN, Clemmer JS, Bergin PF, Xiang L. Obesity and critical illness: insights from animal models. Shock. 2016;45:349-58.

11. Trivedi V, Bavishi C, Jean R. Impact of obesity on sepsis mortality: A systematic review. J Crit Care. 2015;30:518-24.

12. Orr K, Chien P. Sepsis in obese pregnant women. Best Pract Res Clin Obstet Gynaecol. 2015;29:377-93.

13. Huttunen R, Karppelin M, Syrjänen J. Obesity and nosocomial infections. J Hosp Infect. 2013;85:8-16.

14. Labonté MÈ, Couture P, Richard C, Desroches S, Lamarche B. Impact of dairy products on biomarkers of inflammation: a systematic review of randomized controlled nutritional intervention studies in overweight and obese adults. Am J Clin Nutr. 2013;97:706-17.

15. DeBoer MD. Obesity, systemic inflammation, and increased risk for cardiovascular disease and diabetes among adolescents: a need for screening tools to target interventions. Nutrition. 2013;29:379-86.

16. Marques MB, Langouche L. Endocrine, metabolic, and morphologic alterations of adipose tissue during critical illness. Crit Care Med. 2013;41:317-25.

17. Miehsler W. Mortality, morbidity and special issues of obese ICU patients. Wien Med Wochenschr. 2010;160:124-8.

18. McCallister JW, Adkins EJ, O'Brien JM Jr. Obesity and acute lung injury. Clin Chest Med. 2009;30:495-508.

19. Singer G, Granger DN. Inflammatory responses underlying the microvascular dysfunction associated with obesity and insulin resistance. Microcirculation. 2007;14:375-87.

20. Pepper, DJ; Sun, JF; Welsh, J; Cui, XZ; Suffredini, AF; Eichacker, PQ. Increased body mass index and adjusted mortality in ICU patients with sepsis or septic shock: a systematic review and meta-analysis. Crit Care. 2016;20:181.

21. Cave MC, Hurt RT, Frazier TH, Matheson PJ, Garrison RN, McClain CJ. Obesity, inflammation, and the potential application of pharmaconutrition. Nutr Clin Pract. 2008;23:16-34.

22. Neves AL, Coelho J, Couto L, Leite-Moreira A, Roncon-Albuquerque R Jr. Metabolic endotoxemia: a molecular link between obesity and cardiovascular risk. J Mol Endocrinol. 2013;51:R51-64.

**Conference summary: 13**

1. Fried J , Sobelman S , Villatoro G , Grotts J , Paras T , Meller D, et al. Obesity and Mortality in Severe Sepsis and Septic Shock: No "Paradox. Chest. 2015;148:298A.

2. Restrepo MI, Mortensen EM, Sutton S , Bollinger M, Copeland LA , Pugh, MJV , et al. Impact Of Obesity On Mortality For Veterans With Sepsis. Am J Respir Crit Care Med. 2011;183:A3854

3. Wacharasint P, Boyd J, Russell J, Walley K. Obese Patients Have Decreased Mortality and Express a Lower Inflammatory Cytokine Profile During Septic Shock. Chest. 2012;142:411A

4. Youssef J, Xiang Q, Tarima S, Uysal N，Tomic R. Impact Of Obesity On Outcomes In Patients With Septic Shock. Am J Respir Crit Care Med. 185;2012:A4118.

5. Li MJ; Chang HH; Yang YL; Lu MY; Shao PL; Fu CM, et al. Infectious Characteristics And Long-Term Outcome In Obese Pediatric Acute Lymphoblastic Leukemia Patients. Haematologica. 2016;101:484.

6. Moslim M, Augustin T, Brethauer S, Rogers A, Alaedeen D, Aminian A, et al. Obesity and Its Implications on Morbidity and Mortality After Appendectomy in Younger Adults. Gastroenterology. 2016;150:S1185-6.

7. Diederichs T, Unverzagt S, Mueller-Tidow C, Weber T, Mueller LP. Longitudinal decline of body-mass-index (BMI) and low serum albumin correlate with incidence of sepsis and non-relapse mortality (NRM) beyond day+110 after allogeneic hematopoietic stem cell transplantation (SCT). Bone Marrow Transplant. 2015;50:S420.

8. Raymer DS, Vader JM, Nassif ME, Sparrow CT, Larue SJ, Ewald GA. Increased BMI Is Associated with Left Ventricular Assist Device-related Infectious Complications. J Heart Lung Transplant. 2015;33:S197.

9. Castoldi A, Oliveira V, Amano M, Aguiar C, Caricilli A, Vieira P. The Role Of Obesity And Adapter Molecule MyD88 In The Severity Of Acute Kidney Injury Induced By Experimental Sepsis. Nephrol Dial Transplant. 2013;28:408.

10. Singer G, Ruedl R, Sagmeister M, Deutschmann A, Granger DN. Hepcidin and Ferroportin in Different Murine Models of Obesity Challenged with Polymicrobial Sepsis. FASEB J. 2013;27:947.2.

11. Nagel J L, Kunapuli A, Smith J, Bendali-Amor R, Gandhi T, Washer L. Evaluation of Adjusted-Dose versus Full Dose Daptomycin for the Treatment of Vancomycin-Resistant Enterococcal (VRE) Bacteremia in Morbidly Obese Patients. Antimicrob Agents Chemother. 2013;53:K-169.

12. Mannino C, Ajmera A, Giordano S, Willsie P, Peikin S. Body Mass Index (BMI) Does Not Affect Survival and Length of Stay (LOS) in Septic Shock Patients. Am J Gastroenterol. 2011;106:S429-30.

13. Giles KA, Hamdan AD, Pomposelli FB, Wyers MC, Siracuse JJ, Schermerhorn ML. Body Mass Index: Surgical Site Infections and Mortality after Lower Extremity Bypass from the National Surgical Quality Improvement Program 2005-2007. Ann Vasc Surg. 2010;24:48-56.

**Case report: 3**

1. Utrup TR, Mueller EW, Healy DP, Callcut RA, Peterson JD, Hurford WE. High-dose ciprofloxacin for serious gram-negative infection in an obese, critically ill patient receiving continuous venovenous hemodiafiltration. Ann Pharmacother. 2010;44:1660-4.

2. Heather L. Martin, RN, CCRN, CEN. Septic Shock in a Critically Ill Young Man with Morbid Obesity. Bariatr. Nurs. Surg. Patient Care. 2011;6:65-68.

3. Muppala H, Rafi J, Arthur I. Morbidly obese woman unaware of pregnancy until full-term and complicated by intraamniotic sepsis with pseudomonas. Infect Dis Obstet Gynecol. 2007;2007:51689.

**Letter: 3**

1. Abbate LM, Perman SM, Ginde AA, Clambey ET, Van Pelt RE. Age Modifies the Association Between Obesity and Mortality in Individuals Hospitalized with Severe Sepsis. J Am Geriatr Soc. 2016;64:882-3.

2. O'Brien JM. Obesity-related excess mortality rate in an adult intensive care unit: a risk-adjusted matched cohort study. Crit Care Med. 2004;32:1980.

3. Bistrian BR. Effect of prior weight loss on mortality in the critically ill obese. Crit Care Med. 2015;43:e30.

**Editorials: 3**

1. Rivers E. Implementation of an evidence-based "standard operating procedure" and outcome in septic shock: what a sepsis pilot must consider before taking flight with your next patient. Crit Care Med. 2006;34:1247.

2. Rice TW. Obesity in acute lung injury: The "weight" is over. Chest. 2007;131:333-4.

3. Koch, Linda. Effect of maternal obesity on neonatal outcomes. Nat Rev Endocrinol. 2013;9:439.

**Poster presentation: 1**

1. Lee S M, Kang J W, JoY H, Kim K, Lee J H, Lee J, et al. Underweight is associated with mortality in patients with severe sepsis and septic shock. Intensive Care Med Exp. 2015;3 Suppl 1:A876.

**No evidence of sepsis: 28**

1. Martino JL, Stapleton RD, Wang M, Day AG, Cahill NE, Dixon AE, et al. Extreme Obesity and Outcomes in Critically Ill Patients. Chest. 2011;140:1198-206.

2. Soto GJ, Frank AJ, Christiani DC, Gong MN. Body Mass Index and Acute Kidney Injury in the Acute Respiratory Distress Syndrome. Crit Care Med. 2012;40:2601-8.

3. Stewart L, Griffiss JM, Jarvis GA, Way LW. The association between body mass index and severe biliary infections: a multivariate analysis. Am J Surg. 2012;204:574-9.

4. Buehler L, Fayfman M, Alexopoulos AS, Zhao L, Farrokhi F, Weaver J, et al. The impact of hyperglycemia and obesity on hospitalization costs and clinical outcome in general surgery patients. J Diabetes Complications. 2015;29:1177-82.

5. Dossett LA, Dageforde LA, Swenson BR, Metzger R, Bonatti H, Sawyer RG, et al. Obesity and site-specific nosocomial infection risk in the intensive care unit. Surg Infect. 2009;10:137-42.

6. Garrouste-Orgeas M1, Troché G, Azoulay E, Caubel A, de Lassence A, Cheval C, et al. Body mass index. An additional prognostic factor in ICU patients. Intensive Care Med. 2004;30:437-43.

7. Gong MN, Bajwa EK, Thompson BT, Christiani DC. Body mass index is associated with the development of acute respiratory distress syndrome. Thorax. 2010;65:44-50.

8. Hall RG, Blaszczyk AT, Thompson KA, Brouse SD, Giuliano CA, Frei CR, Impact of empiric weight-based vancomycin dosing on nephrotoxicity and mortality in geriatric patients with methicillin-resistant Staphylococcus aureus bacteraemia. J Clin Pharm Ther. 2014;39:653-7.

9. Huttunen R, Laine J, Lumio J, Vuento R, Syrjänen J. Obesity and smoking are factors associated with poor prognosis in patients with bacteraemia. BMC Infect Dis. 2007;9;7:13.

10. Kratz M, Kuzma JN, Hagman DK, van Yserloo B, Matthys CC, Callahan HS, et al. J Nutr. 2013;143:1340-7.

11. Labonte ME, Couture P, Richard C, Desroches S, Lamarche, B. Impact of dairy products on biomarkers of inflammation: a systematic review of randomized controlled nutritional intervention studies in overweight and obese adults. 2013;97:706-17.

13. Langley G, Hao Y, Pondo T, Miller L, Petit S, Thomas A, et al. The Impact of Obesity and Diabetes on the Risk of Disease and Death due to Invasive Group A Streptococcus Infections in Adults. Clin Infect Dis. 2016;62:845-52.

14. Lee SJ, Bose S, Seo JG, Chung WS, Lim CY, Kim H. The effects of co-administration of probiotics with herbal medicine on obesity, metabolic endotoxemia and dysbiosis: a randomized double-blind controlled clinical trial. Clin Nutr. 2014;33:973-81.

15. Lizza BD, Rhodes NJ, Esterly JS, Toy C, Lopez J, Scheetz MH. Impact of body mass index on clinical outcomes in patients with gram-negative bacteria bloodstream infections. J Infect Chemother. 2016;22:671-6.

16. McCallister JW, Adkins EJ, O'Brien JM Jr. Obesity and Acute Lung Injury. Clin Chest Med. 2009;30:495-508.

17. Bercault N, Boulain T, Kuteifan K, Wolf M, Runge I, Fleury JC. Obesity-related excess mortality rate in an adult intensive care unit: A risk-adjusted matched cohort study. Crit Care Med. 2004;32:998-1003.

18. Soubani AO, Chen W, Jang H. The outcome of acute respiratory distress syndrome in relation to body mass index and diabetes mellitus. Heart Lung. 2015;44:441-7.

19. Stewart L, Griffiss JM, Jarvis GA, Way LW. The association between body mass index and severe biliary infections: a multivariate analysis. Am J Surg. 2012;204:574-9.

20. Sturm AW, Allen N, Rafferty KD, Fish DN, Toschlog E, Newell M. Pharmacokinetic analysis of piperacillin administered with tazobactam in critically ill, morbidly obese surgical patients. Pharmacotherapy. 2014;34:28-35.

21. Tafelski S, Yi H, Ismaeel F, Krannich A, Spies C, Nachtigall I. Obesity in critically ill patients is associated with increased need of mechanical ventilation but not with mortality. J Infect Public Health. 2016;9:577-85.

22. van Eijk LT, van der Pluijm RW, Ramakers BP, Dorresteijn MJ, van der Hoeven JG, Kox M. Body mass index is not associated with cytokine induction during experimental human endotoxemia. Innate Immun. 2014;20:61-7.

23. Wardell S, Wall A, Bryce R, Gjevre JA, Laframboise K, Reid JK. The association between obesity and outcomes in critically ill patients. Can Respir J. 2015;22:23-30.

24. Yaegashi M, Jean R, Zuriqat M, Noack S, Homel P. Outcome of morbid obesity in the intensive care unit. J Intensive Care Med. 2005;20:147-54.

25. Marik PE, Doyle H, Varon J. Is Obesity Protective During Critical Illness? An Analysis of a National ICU Database. Crit Care & Shock. 2003;6:156 – 62.

26. Singanayagam A, Singanayagam A, Chalmers JD. Obesity is associated with improved survival in community-acquired pneumonia. Eur Respir J. 2013;42:180-7.

27. Al-Dorzi HM, Al Harbi SA, Arabi YM. Antibiotic therapy of pneumonia in the obese patient: dosing and delivery. Curr Opin Infect Dis. 2014;27:165-73.

28. Cottam DR, Mattar SG, Barinas-Mitchell E, Eid G, Kuller L, Kelley DE, Schauer PR. The chronic inflammatory hypothesis for the morbidity associated with morbid obesity: implications and effects of weight loss. Obes Surg. 2004;14:589-600.

**No evidence of obesity: 3**

1. Adamzik M1, Frey UH, Möhlenkamp S, Scherag A, Waydhas C, Marggraf G, et al. Aquaporin 5 gene promoter--1364A/C polymorphism associated with 30-day survival in severe sepsis. Anesthesiology. 2011;114:912-7.

2. Dinglas VD, Hopkins RO, Wozniak AW, Hough CL, Morris PE, Jackson JC, et al. One-year outcomes of rosuvastatin versus placebo in sepsis-associated acute respiratory distress syndrome: prospective follow-up of SAILS randomised trial. Thorax. 2016;71:401-10.

3. Vadiei N, Daley MJ, Murthy MS, Shuman CS. Impact of Norepinephrine Weight-Based Dosing Compared With Non-Weight-Based Dosing in Achieving Time to Goal Mean Arterial Pressure in Obese Patients With Septic Shock. Ann Pharmacother. 2017;51:194-202.

**Patients divided not as expected: 6**

1. Nguyen AT, Tsai CL, Hwang LY, Lai D, Markham C, Patel B. Obesity and Mortality, Length of Stay and Hospital Cost among Patients with Sepsis: A Nationwide Inpatient Retrospective Cohort Study. PLoS One. 2016;11:e0154599.

2. Sakr Y1, Elia C, Mascia L, Barberis B, Cardellino S, Livigni S, et al. Being overweight or obese is associated with decreased mortality in critically ill patients: a retrospective analysis of a large regional Italian multicenter cohort. J Crit Care. 2012;27:714-21.

3. Gaulton TG, Weiner MG, Morales KH, Gaieski DF, Mehta J, Lautenbach E. The effect of obesity on clinical outcomes in presumed sepsis: a retrospective cohort study. Intern Emerg Med. 2014;9:213-21.

4. Mica L, Vomela J, Keel M, Trentz O. The impact of body mass index on the development of systemic inflammatory response syndrome and sepsis in patients with polytrauma. Injury. 2014;45:253-8.

5. Papadimitriou-Olivgeris M, Aretha D, Zotou A, Koutsileou K, Zbouki A, Lefkaditi A, et al. The Role of Obesity in Sepsis Outcome among Critically Ill Patients: A Retrospective Cohort Analysis. Biomed Res Int. 2016;2016:5941279.

6. Huttunen R, Laine J, Lumio J, Vuento R, Syrjänen J. Obesity and smoking are factors associated with poor prognosis in patients with bacteraemia. BMC Infect Dis. 2007;7:13.

**Without related outcomes: 5**

1. Hodge EK, Hughes DW, Attridge RL. Effect of Body Weight on Hemodynamic Response in Patients Receiving Fixed-Dose Vasopressin for Septic Shock. Ann Pharmacother. 2016;50:816-23.

2. Lam SW, Bauer SR, Cha SS, Oyen LJ. Lack of an effect of body mass on the hemodynamic response to arginine vasopressin during septic shock. Pharmacotherapy. 2008;28:591-9.

3. Radosevich JJ, Patanwala AE, Erstad BL. Norepinephrine Dosing in Obese and Nonobese Patients With Septic Shock. Am J Crit Care. 2016;25:27-32.

4. Wang HE, Griffin R, Judd S, Shapiro NI, Safford MM. Obesity and risk of sepsis: a population-based cohort study. Obesity. 2013;21:E762-9.

5. Levy H, Small D, Heiselman DE, Riker R, Steingrub J, Chen R, et al. Obesity does not alter the pharmacokinetics of drotrecogin alfa (activated) in severe sepsis. Ann Pharmacother. 2005;39:262-7.

**APPENDIX C**

Quality assessment of the included studies using Newcastle-Ottawa Scale.

| Study | Selection | | | | Comparability | Outcome | | |  |
| --- | --- | --- | --- | --- | --- | --- | --- | --- | --- |
|  | Representativeness of the exposed cohort | Selection of the non exposed cohort | Ascertainment of exposure | Demonstration that outcome of interest was not present at start of study | Comparability of cohorts on the basis of the design or analysis | Assessment of outcome | Was follow-up long enough for outcomes to occur | Adequacy of follow up of cohorts | Total score |
| Arabi,2013, | 1 | 1 | 1 | 1 | 2 | 1 | 1 | 1 | 9 |
| Chalkias,2013 | 1 | 1 | 1 | 1 | 0 | 1 | 1 | 1 | 7 |
| Gaulton,2015 | 1 | 1 | 1 | 1 | 2 | 1 | 1 | 1 | 9 |
| Kuperman,2013 | 1 | 1 | 1 | 1 | 2 | 1 | 1 | 1 | 9 |
| Pisitsak,2016 | 0 | 1 | 1 | 1 | 0 | 1 | 1 | 0 | 6 |
| Prescott,2014 | 1 | 1 | 1 | 1 | 2 | 1 | 1 | 1 | 9 |
| Sakr,2008 | 1 | 1 | 1 | 1 | 2 | 1 | 1 | 1 | 9 |
| Wacharasint,  2013 | 1 | 1 | 1 | 1 | 0 | 1 | 1 | 1 | 7 |

**APPENDIX D**


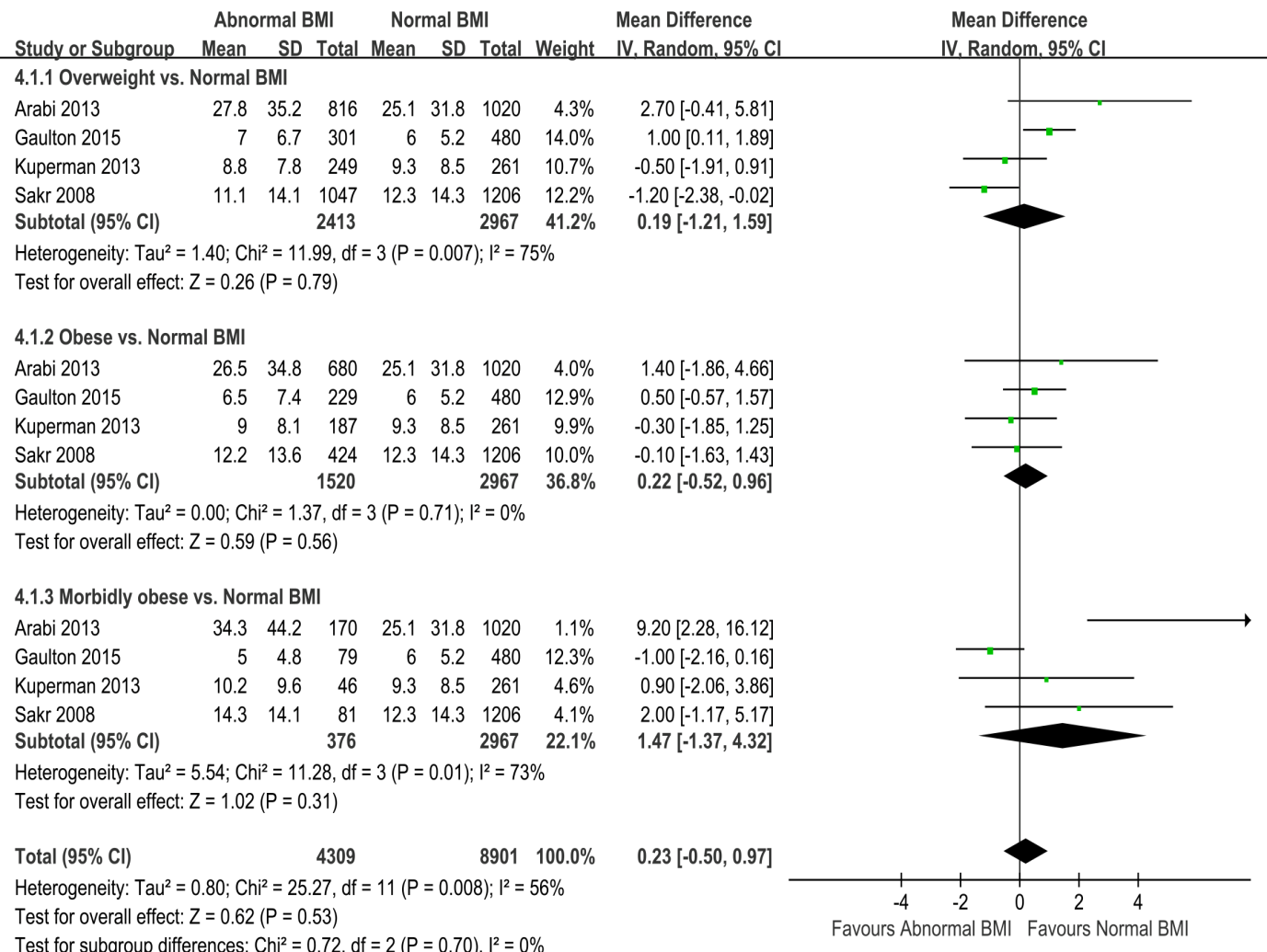


**APPENDIX D**: Effect of BMI on Hospital LOS.

CI confidence interval, BMI body mass index, LOS length of stay

**APPENDIX E**


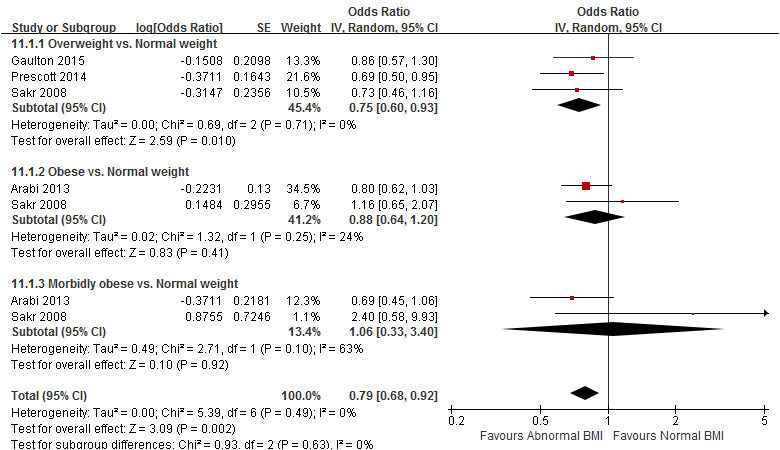


**APPENDIX E:** Adjusted Odds Ratio for Mortality in sepsis.

This figure shows the adjusted effects of different BMI categoried on the odds ratio of mortality reported in studies examining patients with sepsis. Effects for overweight(25<BMI≤29.9) ,obese(30<BMI≤39.9) and morbidly obese(BMI>40) BMIs were calculated compared to patients with normal BMIs(18.5<BMI≤24.9).
